# Domestic gardens and self-reported health: a national population study

**DOI:** 10.1186/s12942-018-0148-6

**Published:** 2018-07-31

**Authors:** Paul Brindley, Anna Jorgensen, Ravi Maheswaran

**Affiliations:** 1 Department of Landscape, University of Sheffield, The Arts Tower, Western Bank, Sheffield, S10 2TN UK; 2 Public Health GIS Unit, School of Health and Related Research, University of Sheffield, Regent Court, 30 Regent Street, Sheffield, S1 4DA UK

**Keywords:** Domestic gardens, Greenspace, General health, UK census, Health inequalities

## Abstract

**Background:**

There is a growing recognition of the health benefits of the natural environment. Whilst domestic gardens account for a significant proportion of greenspace in urban areas, few studies, and no population level studies, have investigated their potential health benefits. With gardens offering immediate interaction with nature on our doorsteps, we hypothesise that garden size will affect general health—with smaller domestic gardens associated with poorer health.

**Methods:**

A small area ecological design was undertaken using two separate analyses based on data from the 2001 and 2011 UK census. The urban population of England was classified into ‘quintiles’ based on deprivation (Index of Multiple Deprivation) and average garden size (Generalised Land Use Database). Self-reported general health was obtained from the UK population census. We controlled for greenspace exposure, population density, air pollution, house prices, smoking, and geographic location. Models were stratified to explore the associations.

**Results:**

Smaller domestic gardens were associated with a higher prevalence of self-reported poor health. The adjusted prevalence ratio of poor self-reported general health for the quintile with smallest average garden size was 1.13 (95% CI 1.12–1.14) relative to the quintile with the largest gardens. Additionally, the analysis suggested that income-related inequalities in health were greater in areas with smaller gardens. The adjusted prevalence ratio for poor self-reported general health for the most income deprived quintile compared against the least deprived was 1.72 (95% CI 1.64–1.79) in the areas with the smallest gardens, compared to 1.31 (95% CI 1.21–1.42) in areas with the largest gardens.

**Conclusions:**

Residents of areas with small domestic gardens have the highest levels of poor health/health inequality related to income deprivation. Although causality needs to be confirmed, the implications for new housing are that adequate garden sizes may be an important means of reducing socioeconomic health inequalities. These findings suggest that the trend for continued urban densification and new housing with minimal gardens could have adverse impacts on health.

**Electronic supplementary material:**

The online version of this article (10.1186/s12942-018-0148-6) contains supplementary material, which is available to authorized users.

## Background

There is a growing evidence base demonstrating health and wellbeing benefits from exposure to natural environments (often referred to generically as ‘greenspace’). In contrast and despite their prevalence, the role of domestic gardens remains unclear, with relatively few studies, and no population level studies, exploring their contribution for health. This research seeks to address this disparity.

Domestic gardens contribute a large proportion of the total urban area (for example, 23% in Sheffield, UK [1] and 36% in Dunedin, New Zealand [2]). Coupled with this is the trend over time which has seen increases in the development of garden space into domestic and other uses (e.g. house extensions and new dwellings). In England over the four-year period since 2013, over 4600 hectares of garden were converted to other uses [3]. Despite their widespread prevalence, domestic gardens are, however, surprisingly under-researched [4]. This is most likely due to their heterogeneity and lack of available secondary data for these frequently small and private spaces. Many studies of health and greenspace do not include domestic gardens. Frequently, private gardens are either aggregated with all other greenspace measures [5, 6]; combined within an urban category [7, 8]; or excluded from analysis entirely [9–12].

The reported health benefits of greenspace more generally are diverse—including reducing obesity; promoting mental health (for example by reducing the risk of stress, tendency to psychiatric morbidity, psychological distress, depressive symptoms, clinical anxiety, depression and mood disorders); affecting birth outcomes; educational performance and academic attainment; influencing physiological health (for example cancer, diabetes, cardiovascular outcomes); improving general health; and ultimately affecting mortality [8, 9, 13–15]. Furthermore, it has been suggested that health inequalities are worse in areas with less greenspace [9]. Proposed possible salutogenic mechanisms include: physical activity; social contact; psychological pathways (stress, cognitive, affective); reduced air pollution; and immunological function/regulation [8].

The potential health effects arising from domestic gardens may be the same as those outlined above. However, they may also have a distinctive role. Gardens, due to their close proximity to the home, provide the opportunity for people to have an immediate and sustained contact with nature [1]. Residents have an autonomy over the garden and a level of privacy which they cannot possess in public greenspaces [16]. There is also a symbiosis between the garden as a physical space and the activity of gardening. Furthermore, evidence suggests that spending time in the garden is associated with increased perceptions of social cohesion between neighbours [17]. Importantly, people who lack a private garden do not compensate with more frequent visits to public greenspaces [18].

### Health benefits of domestic gardens

The evidence for the health benefits of domestic gardens remains mixed and inconclusive. No statistical difference was found by two studies investigating the relationship between greenspace and mental health when analysis was repeated including and excluding domestic gardens from their total greenspace measures [5, 6]. In a study of the association between greenspace and perceived general health, some analyses demonstrated a positive health effect associated with having a garden, but in others the effects were not significant [19]. A recent study of the North West of England found that in urban areas the proportion of land classified as domestic gardens in Lower-layer Super Output Areas (LSOA: a geographic unit commonly used for reporting small area statistics in England containing an average population of approximately 1500) was more closely associated with lower levels of health deprivation (as measured by the English index of multiple deprivation) than the proportion of land classified as greenspace [20].

Whilst the effect of domestic gardens upon mood or anxiety remains uncertain [21, 22], there is support for gardens reducing stress [21, 23–27]. It seems likely, however, that contact with nature in domestic gardens leads to both hedonic (positive emotional states) and eudaimonic (meaning of life) wellbeing benefits associated with a sense of nature connectedness [28]. There is also strong evidence concerning the health benefits of gardening, as an activity. A recent review found support for gardening improving physical and mental health, and social wellbeing [29].

Any benefits may not be universal, and the type of garden is likely to be critical [30]. The size of the garden and diversity of features (e.g. a lawn, water, and so forth) were associated with increases in perceived restorativeness (recovery in ability to concentrate) [31]. People with larger gardens were more likely to have increased tree cover and spend more time in the garden [32], which might contribute to enhanced health benefits.

Potential hypothesised pathways between average garden size and poor general health could be categorised as:

- Gardening [29]: areas with larger average gardens might contain populations that are more likely to have increased levels of gardening;
- Other individual/household level exposure: individuals with access to larger gardens might derive positive health benefits related to the size of the garden, for example: increased time spent within larger gardens [32]. Other mechanisms include enhanced potential to undertake physical exercise [33] within larger gardens; physiological benefits from views [34] within their own garden—possibly related to larger gardens having a greater diversity of garden features [31] or increased tree coverage [32]; an enhanced feeling of ‘being away’—a key characteristic of psychologically restorative environments [35]; physiological benefits from increased tranquillity (and reduced noise levels) [36] or enhanced biodiversity [37, 38] associated with larger gardens.
- Population level impact: benefits may accrue from living in an area of larger gardens even if the individual(s) themselves do not have access to a large garden, for example through reduced air pollution [34], more regulated temperatures [39], physiological benefits from views [34] of other people’s gardens, or enhanced biodiversity in the area in general [37, 38].

Our hypothesis is that domestic gardens may have a beneficial effect on general health and specifically that areas with smaller gardens may exhibit higher levels of poor self-reported general health (even after accounting for differences in socio-economic characteristics, such as deprivation).

## Methods

### Study area

The geographical area for the study was England. We used LSOAs as the spatial units for analysis. Rural areas are likely to have close proximity to a wide range of natural environments. However, we wanted to focus on households whose domestic garden potentially offers the most immediate contact with nature. According to the 2011 Census of Population 82.4% of England’s population live in urban areas. For these reasons only those LSOAs defined as urban (by the UK Office for National Statistics’ 2001 Rural–Urban Classification) were used in our analysis.

Our data included Census of Population for two time periods: 2001 and 2011. Therefore, we included all 26,455 urban LSOAs in 2001 but could only include a subset of urban LSOAs in 2011. This was because LSOA boundaries changed during the period. Some LSOAs were split while others were merged to take account of population changes and new developments. We used 25,766 urban LSOAs in 2011 (95% of urban LSOAs in 2011) which remained unchanged in order to maintain comparability between analyses based on the two censuses.

### Study design

This study implemented a population (ecological) study design using routinely accessible secondary datasets. We examined the association between average domestic garden size and self-reported general health in urban census areas in England. Furthermore, we explored the health-inequalities associated with varying garden size.

### Data

Self-reported general health was obtained from the UK census in both 2001 and 2011 at the LSOA scale. The use of the two time periods was to explore if patterns were consistent, thus adding a degree of robustness to findings. A number of studies have shown that self-reported general health are a reliable measure of objectively measured health outcomes [40–42]. In 2001 people were asked to assess whether their health was good, fairly good or not good. In 2011 the question was asked on a five-point scale: very good, good, fair, bad or very bad. Two separate independent measures of poor health were constructed using (1) the ‘not good’ health category from the 2001 census and (2) the aggregation of ‘bad’ and ‘very bad’ health categories from the 2011 census.

Indirect standardisation was undertaken for broad age (0–15; 16–34; 35–49; 50–59; 60–64; 65–84; and over 84) and sex categories. These data formed the dependent variables for our two models (Model One: 2001 health data; Model Two: 2011 health data). Self-reported general health from the census has been used within a number of similar population (ecological) studies [8, 12].

Domestic gardens and greenspace measurements were obtained from the Generalised Land Use Database (GLUD). GLUD is a national classification developed by the Office for National Statistics which allocates all identifiable land features on Ordnance Survey MasterMap into simplified categories. MasterMap for the whole country is an extremely large digital database and the simplified classification has transformed it for use in country wide statistical analyses.

GLUD provides the total area for nine land use categories in every English LSOA. The land categories are: domestic buildings, non-domestic buildings, roads, paths, rail, gardens (domestic), greenspace, water, other land uses. We used both the total greenspace and domestic garden measures from GLUD. The garden extent was converted into an average garden size measurement by dividing the total garden size in the LSOA by the number of households recorded by the census. GLUD is only available for the years 2000 and 2005. We used the 2000 GLUD to compare against the 2001 health data and the 2005 GLUD for the 2011 health data. Due to GLUD 2005 relating to 2001 LSOA boundary definitions only those LSOAs whose boundaries did not change in the period 2001–2011 were used within analysis for Model Two. A number of similar studies have used GLUD as their source of land cover data [8, 9, 12, 20].

We adjusted for area characteristics that were plausibly associated with general health. The income, employment and education domains of the English Index of Multiple Deprivation (EIMD) were used, for 2004 (the first EIMD available for LSOAs, for comparison with the 2001 health data) and 2010 (the closest time period to the 2011 health data). All three EIMD domains have commonly been collectively used to adjust for socio-economic deprivation [8, 9, 12].

Due to potential associations with general health, we also controlled for the levels of pollution, smoking, population density, house price and geographic region. Pollution data consisted of 1 km gridded estimates of Particulate Matter of ten microns in diameter or smaller (PM_10_) modelled by the UK’s Department for Environment, Food and Rural Affairs in the years 2004 and 2010 and assigned to LSOAs by the population weighted average for each LSOA (where the population represented the census headcounts at unit postcode level). A proxy for smoking was obtained using the number of lung cancer hospital admissions for the period 1st April 2002 to 31st March 2014 [43]. The ratio of observed to expected counts was calculated for each LSOA, with expected counts adjusted for age and sex. The same smoking proxy data were used in both models. Population density was calculated for each LSOA in 2001 and 2011, as the resident population from the census divided by the LSOA area. The average house price at the LSOA level was generated from HM Land Registry Price Paid Data for the years 2004 and 2010. A standard z-score method was implemented to adjust for differences in house type (detached, semi-detached, terraced, flat and other) and geographic district. The nine Regions of England were included to account for any geographic differences at this scale. These confounders have been commonly included in numerous similar greenspace ecological studies [9, 10, 12, 44].

All independent data were classified into quintiles. The average garden size within the LSOAs (hectares) resulted in the following quintiles: 0.00–0.009; 0.010–0.017; 0.018–0.021; 0.022–0.029; and 0.030–0.233.

### Analysis

Negative binomial regression was used to test whether there was an independent association between average domestic garden size and self-reported poor health, after controlling for the confounding factors previously described. The dependent variable was the total number of people reporting poor health, whilst the offset was the number expected given the age and sex composition (indirect standardisation). Poisson models were rejected due to over dispersion. Analysis was undertaken within SAS version 9.4.

In addition to the main analysis described above, we also explored whether the association between poor self-reported general health and deprivation varied by average garden size (utilising the approach of previous similar work [9]). This was achieved using a sequence of models stratified by the average garden size quintile (for example the first model explored the relationship between deprivation quintile and general health in the quintile with the smallest average garden sizes, the second model explored the same relationship for people in the second smallest average garden size quintile, and so forth). Models were adjusted for the same confounders as previously described.

### Sensitivity analysis

The following sensitivity analysis was undertaken to ensure robustness of our findings. Separate exploratory analysis was undertaken for each age band within Model One (0–15; 16–34; 35–49; 50–59; 60–64; 65–84; and 85 and over). To confirm that associations between average garden size and socio-economic status were accounted for (in addition to the house price and deprivation variables within the main models), average income at the Middle-layer Super Output Area (MSOA) level (2004/2005) and the most frequent ACORN classification (a postcode-level consumer classification that segments the UK population based on a plethora of household and individual level data) within the LSOA were also analysed.

Testing also included replacing the hospital admissions for lung cancer variable used in the main models with modelled estimated smoking prevalence at the MSOA scale (2003–2005 data for Model One and 2006–2008 for Model Two). This was undertaken to assess the robustness of the hospital admissions data as a proxy for smoking. Finally, the proportion of LSOA area consisting of garden (from GLUD) was used as a variable instead of the average garden size in case estimates were influenced by converting our garden metric to an average measurement.

## Results

Table 1 presents means (SD) for variables used in Model One. The 26,455 LSOAs in English urban areas included in the study covered 81% of the total population of England.

**Table 1:** Characteristics of English urban lower-layer super output areas used within model one

Variable	Mean	SD
Average domestic garden size within the LSOA (hectares), 2000/2001	0.02	0.01
% of LSOA covered by domestic gardens [as used within sensitivity analysis] from GLUD 2000	29.06	15.93
% of LSOA covered by total greenspace (excludes gardens) from GLUD 2000	33.77	24.32
IMD Income score for the LSOA, 2004	0.15	0.12
IMD Education score for the LSOA, 2004	0.11	0.08
IMD Employment score for the LSOA, 2004	23.68	19.53
Average house price Z-score for LSOAs, 2004	− 0.09	0.54
Population density within the LSOA, 2001 (people per hectare)	47.19	38.57
Average pollution from particular matter (of ten microns in diameter or smaller) in the LSOA, 2004	21.79	2.97
Ratio of observed to expected lung cancer hospital admissions (01/04/2002–31/03/2014)	1.07	0.82
The percentage of people reporting ‘poor health’ from the 2001 Census of Population at the LSOA level	8.90	3.42

**Fig. 1:**
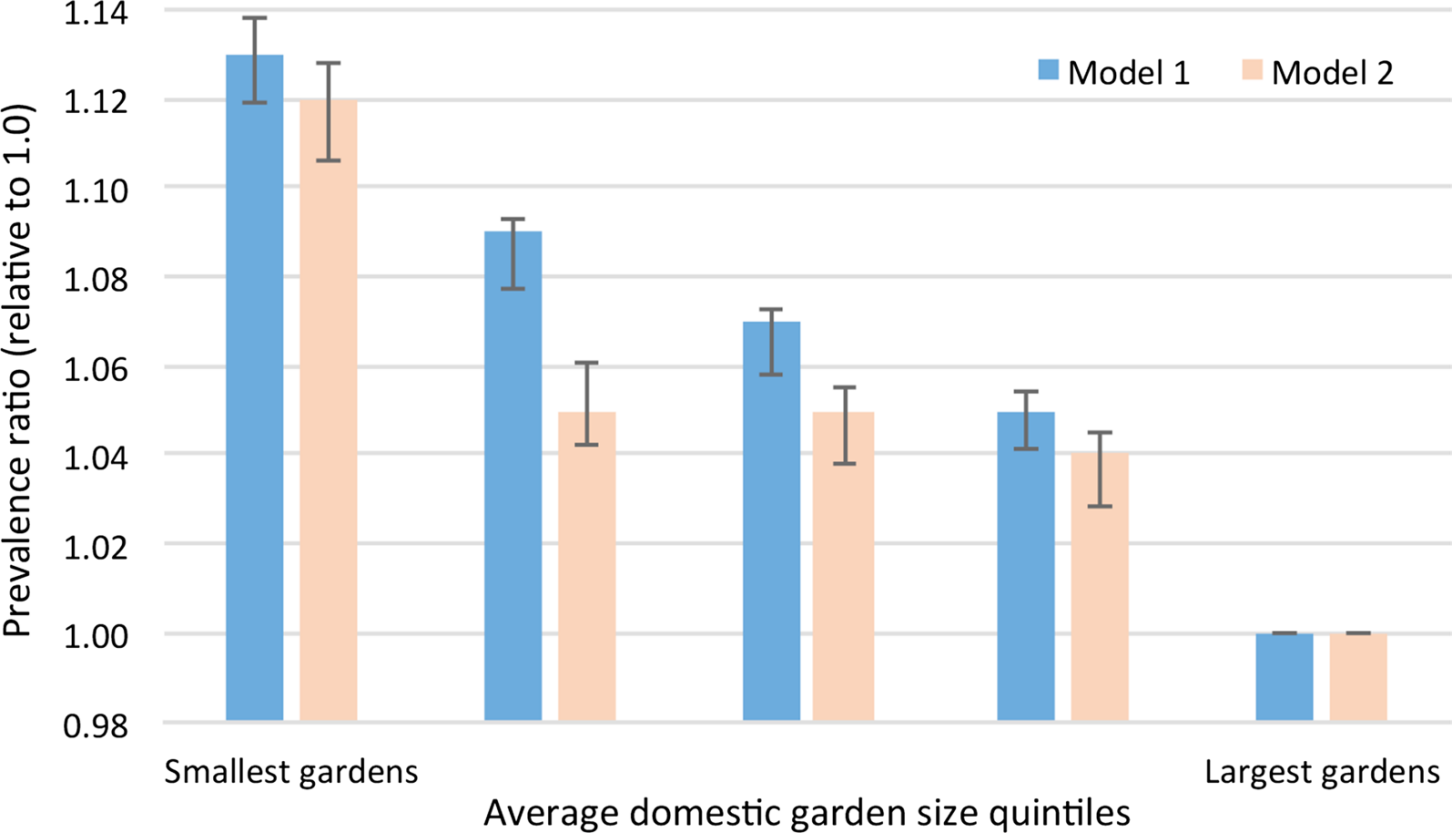
Strength of association between general health and average garden size, accounting for confounders (with 95% CI)

The independent relationship between average domestic garden size and self-reported poor general health, after controlling for confounding factors and deprivation, is shown in Fig. 1. It clearly shows a higher adjusted prevalence ratio for populations residing in areas of smaller average garden size. The adjusted prevalence ratio for poor health in Model One was 1.13 (95% CI 1.12–1.14) for the quintile with the smallest average garden sizes compared against the quintile with the largest average gardens, when accounting for deprivation and confounders. A similar ratio was evident for the later time period (Model Two) where the adjusted prevalence ratio was 1.12 (95% CI 1.11–1.13) for the same quintiles.

Table 2 contains the prevalence ratios for all variables for both models. Whilst average garden size appears to be playing an important role, the effects of total greenspace upon poor health (when accounting for confounders) are not evident. A significant positive relationship between greenspace and poor health only transpired if the average garden variable was removed—and the effect was modest (adjusted prevalence ratio of 1.03, 95% CI 1.02–1.03—data not shown). This finding is considered further within the discussion. There was no significant interaction between average garden size and total greenspace (p = 0.251).

**Table 2:** Regression results: association between general health and modelled output

Variable	Quintile	Model one		Model two	
		Adjusted prevalence ratio	95% CI	Adjusted prevalence ratio	95% CI
Total greenspace	1 Least green	0.99	0.977, 0.995	0.99	0.983, 1.005
	2	0.98	0.976, 0.993	0.99	0.982, 1.002
	3	0.99	0.979, 0.995	1.00	0.987, 1.006
	4	0.99	0.978, 0.992	1.00	0.989, 1.007
	5 Most green	1		1	
Average domestic garden size	1 Smallest gardens	1.13	1.119, 1.138	1.12	1.106, 1.128
	2	1.09	1.077, 1.093	1.05	1.042, 1.061
	3	1.07	1.058, 1.073	1.05	1.038, 1.055
	4	1.05	1.041, 1.054	1.04	1.028, 1.045
	5 Largest gardens	1		1	
Income deprivation	1 Most deprived	1.44	1.422, 1.461	1.69	1.659, 1.715
	2	1.30	1.281, 1.310	1.43	1.413, 1.453
	3	1.21	1.197, 1.219	1.29	1.276, 1.305
	4	1.11	1.105, 1.121	1.15	1.143, 1.164
	5 Least deprived	1		1	
Employment deprivation	1 Most deprived	1.45	1.429, 1.465	1.55	1.522, 1.570
	2	1.29	1.280, 1.306	1.36	1.341, 1.376
	3	1.19	1.180, 1.199	1.24	1.228, 1.256
	4	1.11	1.098, 1.114	1.14	1.135, 1.155
	5 Least deprived	1		1	
Education deprivation	1 Most deprived	1.23	1.215, 1.240	1.31	1.294, 1.327
	2	1.17	1.157, 1.177	1.23	1.214, 1.240
	3	1.13	1.123, 1.140	1.17	1.161, 1.183
	4	1.09	1.082, 1.096	1.11	1.106, 1.124
	5 Least deprived	1		1	
Population density	1 Highest density	1.01	1.002, 1.024	1.04	1.026, 1.053
	2	1.00	0.995, 1.014	1.01	1.000, 1.022
	3	1.01	0.998, 1.015	1.01	1.000, 1.020
	4	1.01	1.002, 1.016	1.00	0.994, 1.011
	5 Lowest density	1		1	
Pollution (PM_10_)	1 Highest pollution	1.08	1.076, 1.093	1.08	1.065, 1.087
	2	1.05	1.040, 1.056	1.05	1.044, 1.061
	3	1.04	1.036, 1.050	1.05	1.038, 1.054
	4	1.04	1.030, 1.043	1.03	1.026, 1.041
	5 Lowest pollution	1		1	
Smoking proxy: lung cancer hospital admissions (2002–2014)	1 Highest ‘smoking’	1.03	1.018, 1.032	1.04	1.034, 1.051
	2	1.02	1.016, 1.029	1.03	1.024, 1.040
	3	1.02	1.012, 1.024	1.02	1.016, 1.031
	4	1.01	1.007, 1.019	1.02	1.015, 1.030
	5 Lowest ‘smoking’	1		1	
Average house prices	1 Lowest prices	1.03	1.027, 1.042	1.06	1.046, 1.064
	2	1.02	1.017, 1.030	1.04	1.031, 1.047
	3	1.02	1.016, 1.029	1.03	1.022, 1.038
	4	1.02	1.009, 1.021	1.02	1.016, 1.031
	5 Highest prices	1		1	
Region of England	East Midlands	0.95	0.938, 0.955	0.97	0.956, 0.977
	East of England	0.87	0.857, 0.873	0.88	0.875, 0.893
	London	0.90	0.887, 0.904	0.96	0.945, 0.966
	North East	1.03	1.020, 1.041	1.08	1.065, 1.092
	North West	1.04	1.029, 1.044	1.09	1.078, 1.098
	South East	0.87	0.859, 0.873	0.90	0.891, 0.908
	South West	0.92	0.914, 0.930	0.94	0.934, 0.954
	West Midlands	0.96	0.952, 0.967	1.00	0.994, 1.013
	Yorkshire and The Humber	1		1	

**Fig. 2:**
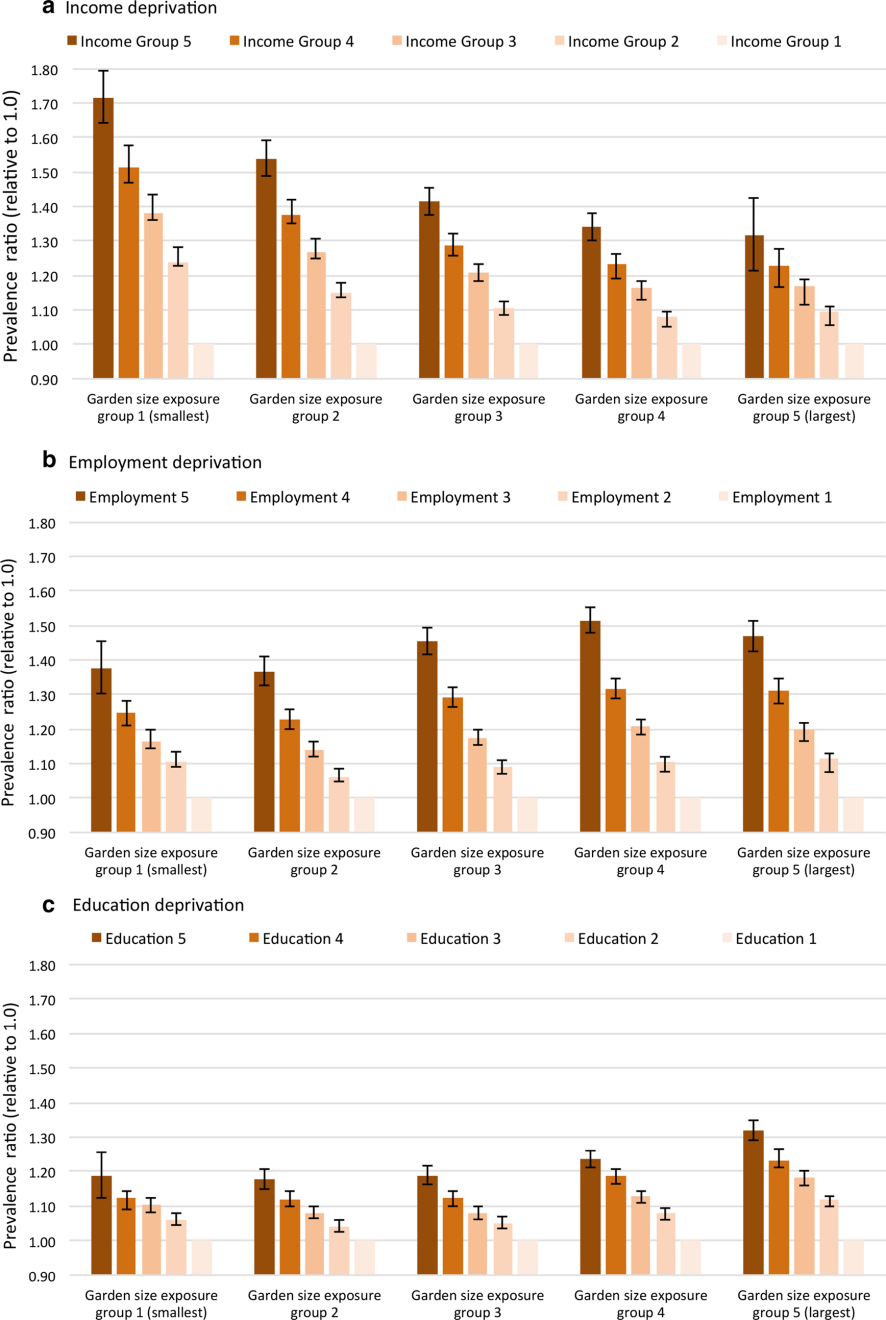
Prevalence ratios for general health in deprivation quintiles (relative to income group 1—least deprived), stratified by average garden size (with 95% CI)

Significant interaction was found between average garden size and all three-deprivation terms (p values < 0.001). Exploring health inequalities, Fig. 2a highlights the interaction between income-deprivation and average garden size for Model One. The adjusted prevalence ratio for self-reported poor health for the most income-deprived quintile versus the least deprived was 1.72 (95% CI 1.64–1.79) in the quintile with the smallest average garden size, whereas it was 1.31 (95% CI 1.21–1.42) in the quintile with the largest average gardens. A similar pattern was evident when testing interactions using data from Model Two (data not shown). It should, however, be noted that similar interactions are not present for the other deprivation domains (employment—Fig. 2b, or education—Fig. 2c).

### Sensitivity analysis results

Separate exploratory analysis for each age and sex band (Additional file 1: Table S1) demonstrated that the association between garden size and poor self-reported general health was generally consistent through age and sex. The effect of garden size was not statistically significant for poor general health of females aged 0–15, 16–34, and 85 and over. Effects were strongest for ages 35–49 and 50–59 (regardless of sex).

The stability of the relationship between garden size and poor health was maintained when additional socio-economic variables were added (Additional file 1: Table S2). This sensitivity analysis was undertaken to ensure that the association was not unduly influenced by garden size acting as a proxy for other socio-economic characteristics (such as income). The similarity of output between both the average garden size (Models One and Two) and the proportion of the LSOA that is garden (sensitivity analysis—Additional file 1: Table S2d) is encouraging and demonstrates robustness.

## Discussion

This is the first national population study to explore the relationship between domestic garden size and health. Our results support our hypothesis that there is an association between health and average domestic garden size. Furthermore, it suggests that income-related inequalities in poor self-reported health are greater in areas with smaller average gardens. Our work should act as a motivation for future studies in this area.

Published work has established strong support for the health benefits of greenspace. Whilst there are fewer studies specifically focussing on the role of gardens; our study supports the notion of health benefits accruing from gardens. The strongest evidence from existing literature concerns their psychological effects through restorativeness and stress reduction [21, 23–26], although a recent review of gardening was able to support a link with physical and mental health, and social wellbeing [29].

Whilst existing evidence has demonstrated the powerful relationship between greenspace and health inequalities in terms of mortality [9], our study was able to find similar support for self-reported general health inequalities relative to average garden size and income deprivation. The results for employment and educational deprivation, however, were less clear. This said, the pattern of the relationship (for both employment and educational deprivation) between the socio-economic category and each garden size was consistent.

Whilst we acknowledge that some ecological studies have found stronger health benefits from greenspace [9], in common with other studies [8] we report a relatively modest effect (when accounting for a wide range of confounders). Our findings corroborate the previously cited recent study in which domestic gardens appear to mitigate poor health more effectively than greenspace [20]. Whilst both studies utilise natural environment data from GLUD (as used extensively in the literature) [8, 9, 12, 20], it is important to note that such data makes no distinction between different types of greenspace and no allowance for varying greenspace quality. This modest effect for greenspace must also be taken within the context of our study’s aims, which are centred on the role of gardens and not greenspace per se. The function of the greenspace variable within our model was to account for geographic differences in greenspace coverage rather than to specifically explore the health effects of greenspace. Our paper demonstrates, however, that the role of domestic gardens is likely to be at least as important as greenspace for influencing general health.

Building on existing studies, for example demonstrating psychological benefits associated with increasing species richness (biodiversity) of urban greenspaces [37], further work is required to investigate the possible effects of quality of garden space on health.

Our population study draws on the power of large datasets to investigate the relationship between garden size and poor health and to assess how this association varied between areas with differing socio-economic status. It used robust health data from a reliable source. The study was hypothesis driven and based upon the existing literature.

Like any ecological, small-area study of this nature, there are a number of limitations. Firstly, correlation does not necessarily imply causation. Whilst we have found a clear association, our study cannot confirm whether there is a causal link. Testing causation would require a range of further work, including cohort studies. The garden size measure might be associated with other risk factors that are not controlled for within our models. Average garden size is likely to be strongly associated with socio-economic position. Whilst sensitivity analysis has been undertaken to explore this possibility, and we have adjusted for socio-economic and other potential confounders, residual or unmeasured confounding could potentially explain the observed link. It is argued, however, that stratified studies like this one, offer the best possible protection against the effects of residual cofounding in ecological investigations [9]. Our findings do not highlight which of the proposed mechanisms through which garden size might influence health and further work is required to explore this.

Secondly, the role of the ecology fallacy should be considered. Given that associations are based on data aggregated to bounded units (in this instance LSOAs), one should not presume that the same associations will hold at the individual level. This is important given that an individual’s garden size may or may not correspond with the average garden size in their LSOA (considered further below).

Thirdly, whilst our study analyses two separate time periods (Model One and Two), there is no means of knowing the extent to which individuals’ garden size may have changed—affecting their longer-term general health.

There are a number of other limitations specific to the work presented here. There have been changes within data throughout the study period. Most notably the questions concerning general health in the UK Census have been modified. In 2001 the question related to period prevalence—asking residents about their health over the last 12 months—whilst in 2010 the measure was one of point prevalence. The three category question in 2001 became five categories. Whilst the EIMD (2004–2010) was generally unchanged, certain variables and weightings were altered. Our study, however, is not concerned with trends and treats each model independently. The fact that both models produce relatively similar output, despite these changes, is encouraging and supports robustness within the models.

Whilst our measure of average garden size is both simple and easily interpretable, it is not without limitation. Primarily, it pays no regard to the distribution (or variety) of garden sizes within the area. The difficultly here is that data (such as GLUD) only provide aggregates (for example to LSOAs) and do not contain information for individual plots. Therefore, two LSOAs may have the same average garden size but may contain very different patterns of garden exposure across their populations. In part, this was the rationale behind also including an alternative garden measure (using the proportion of the LSOA area consisting of garden) within the sensitivity analysis to check the robustness of our findings (see Additional file 1: Table S2d). The similarity between output for both variants is encouraging. Preference for the average garden size measure remains because the proportion of LSOA that is garden will potentially be influenced by the denominator—depending on the total size of the LSOA. Further work is needed to explore alternative garden measures that might potentially reflect the distribution of garden sizes.

It is possible that including lung cancer as a confounder could inadvertently over-adjust effects, as people with lung cancer may be more likely to self-report their general health as poor. Given the relatively small numbers involved at the LSOA scale and the relatively modest adjusted prevalence ratios associated with the lung cancer proxy (see Table 2), however, the potential effects of over-adjustment are likely to be small.

Whilst the two separate models provide indications for different time periods—data are not always available for directly comparable periods. GLUD was only available for the years 2000 and 2005. Associating the 2005 GLUD data to 2011 health data leaves a considerable but unavoidable time gap. For this reason, we should be less confident in the findings of Model Two. Given the similarity between the model output, however, the results are encouraging. Whilst current work focuses on general health, further investigations are required that account for the quality of domestic gardens and to explore the association with mortality.

## Conclusions

In conclusion, our findings demonstrate the association between average domestic garden size and self-reported general health, which persist even when accounting for a multitude of socio-economic confounders. We have been able to show differences in health inequalities between populations exposed to similar levels of income deprivation but who reside in areas of different average garden size. The relationship between these health inequalities and the domains of employment or education deprivation are, however, less transparent and require further investigation.

Whilst affected by the limitations associated with any ecological design, the paper contributes to the early stage evidence base for this topic and clearly demonstrates the requirement for follow-up research. Given the diversity within domestic gardens, further work exploring the different components of gardens that may provide health benefits, alongside the mechanisms by which gardens and garden size may affect health benefits, are important areas of future research.

Even though causality needs confirmation, our work suggests that domestic garden size should be taken into account when planning new housing, as once built, there will be little scope for making changes. This is against a backdrop of continuing urban densification in which there remains little or no incentive for housing developers to provide larger domestic gardens. The potential health effects of domestic gardens need to be seriously taken on-board by planners and policy makers alike. Garden size might be an important factor to help alleviate poor general health.

## Additional file

**Additional file 1.** Supplementary tables.
