# Supplementary material for: Domestic gardens and self-reported health: a national population study
Source: Int J Health Geogr. 2018 Jul 31;17:31. doi: 10.1186/s12942-018-0148-6 (PMC6069855; doi:10.1186/s12942-018-0148-6)
Supplement: Supplementary file 1 — Additional file 1. Supplementary tables. [file 12942_2018_148_MOESM1_ESM.docx]

**ADDITIONAL FILE TABLES:**

**Table S1: Sensitivity analysis regression coefficients for general health and average garden size (accounting for confounders, Model One), stratified by age and sex (95% CI in brackets)**

| **a) Males** |  |  |  |  |  |  |  |
| --- | --- | --- | --- | --- | --- | --- | --- |
| **Average garden size** | **Age: 0-15** | **Age 16-34** | **Age 35-49** | **Age 50-59** | **Age 60-64** | **Age 65-84** | **Age 85 and over** |
| 1 (smallest) | 1.11  (1.02, 1.21) | 1.08  (1.04, 1.12) | 1.23  (1.20, 1.26) | 1.21  (1.19, 1.24) | 1.14  (1.11, 1.17) | 1.15  (1.13, 1.17) | 1.08  (1.01, 1.14) |
| 2 | 1.04  (0.96 – 1.12) | 1.05  (1.02, 1.09) | 1.13  (1.10, 1.15) | 1.14  (1.11, 1.16) | 1.11  (1.08, 1.14) | 1.11  (1.10, 1.13) | 1.06  (1.01, 1.12) |
| 3 | 1.02  (0.95 – 1.10) | 1.00  (0.97, 1.03) | 1.10  (1.08, 1.12) | 1.10  (1.08, 1.12) | 1.11  (1.08, 1.13) | 1.09  (1.08, 1.10) | 1.06  (1.01, 1.11) |
| 4 | 0.97  (0.91 – 1.04) | 0.98  (0.95, 1.01) | 1.05  (1.03, 1.07) | 1.07  (1.05, 1.09) | 1.07  (1.05, 1.10) | 1.07  (1.05, 1.08) | 1.08  (1.03, 1.13) |
| 5 (largest) | 1 | 1 | 1 | 1 | 1 | 1 | 1 |
|  |  |  |  |  |  |  |  |
| **b) Females** |  |  |  |  |  |  |  |
| **Average garden size** | **Age: 0, 15** | **Age 16, 34** | **Age 35, 49** | **Age 50, 59** | **Age 60, 64** | **Age 65, 84** | **Age 85 and over** |
| 1 (smallest) | 1.14  (1.03, 1.26) | 1.02  (0.98, 1.05) | 1.21  (1.18, 1.23) | 1.19  (1.16, 1.21) | 1.15  (1.11, 1.19) | 1.12  (1.10, 1.13) | 0.98  (0.95, 1.01) |
| 2 | 1.08  (0.99, 1.18) | 1.02  (0.99, 1.05) | 1.12  (1.10, 1.14) | 1.12  (1.10, 1.14) | 1.10  (1.07, 1.14) | 1.09  (1.07, 1.10) | 0.99  (0.96, 1.01) |
| 3 | 1.05  (0.97, 1.14) | 1.00  (0.97, 1.03) | 1.09  (1.07, 1.11) | 1.10  (1.08, 1.12) | 1.09  (1.05, 1.12) | 1.07  (1.06, 1.09) | 0.99  (0.97, 1.02) |
| 4 | 1.03  (0.96, 1.11) | 1.00  (0.97, 1.02) | 1.07  (1.05, 1.09) | 1.06  (1.05, 1.08) | 1.07  (1.04, 1.10) | 1.06  (1.05, 1.07) | 1.01  (0.98, 1.03) |
| 5 (largest) | 1 | 1 | 1 | 1 | 1 | 1 | 1 |

**Table S2: Sensitivity analysis regression coefficients for general health and average garden size (accounting for confounders), with additional confounders (a) the most frequent ACORN socio-economic classification group; (b) income at the MSOA scale; (c) alternative smoking variable; (d) alternative garden size variable**

| **a) additional confounder: ACORN majority** | | | | | | |
| --- | --- | --- | --- | --- | --- | --- |
| Average garden size | **Model One:** | |  | **Model Two:** | |  |
|  | adjusted prevalence ratio | CI - upper | CI - lower | adjusted prevalence ratio | CI - upper | CI - lower |
| 1 (smallest) | **1.12** | 1.11 | 1.13 | **1.11** | 1.10 | 1.12 |
| 2 | **1.07** | 1.06 | 1.08 | **1.05** | 1.04 | 1.06 |
| 3 | **1.05** | 1.04 | 1.06 | **1.04** | 1.03 | 1.05 |
| 4 | **1.03** | 1.03 | 1.04 | **1.03** | 1.02 | 1.04 |
| 5 (largest) | **1.00** | 1.00 | 1.00 | **1.00** | 1.00 | 1.00 |
|  |  |  |  |  |  |  |
| **b) additional confounder: Income - Average Weekly Household Net Income Estimate (equivalised after housing costs)** | | | | | | |
| Average garden size | **Model One:** | |  |  |  |  |
|  | adjusted prevalence ratio | CI - upper | CI - lower |  |  |  |
| 1 (smallest) | **1.13** | 1.12 | 1.13 |  |  |  |
| 2 | **1.08** | 1.08 | 1.09 |  |  |  |
| 3 | **1.07** | 1.06 | 1.07 |  |  |  |
| 4 | **1.05** | 1.04 | 1.05 |  |  |  |
| 5 (largest) | **1.00** | 1.00 | 1.00 |  |  |  |
|  |  |  |  |  |  |  |
| **c) additional confounder: Smoking prevalence at the MSOA scale (replacing smoking proxy variable)** | | | | | | |
| Average garden size | **Model One:** | |  | **Model Two:** | |  |
|  | adjusted prevalence ratio | CI - upper | CI - lower | adjusted prevalence ratio | CI - upper | CI - lower |
| 1 (smallest) | **1.13** | 1.12 | 1.14 | **1.09** | 1.08 | 1.11 |
| 2 | **1.08** | 1.08 | 1.09 | **1.04** | 1.03 | 1.05 |
| 3 | **1.07** | 1.06 | 1.07 | **1.04** | 1.03 | 1.04 |
| 4 | **1.05** | 1.04 | 1.05 | **1.03** | 1.02 | 1.04 |
| 5 (largest) | **1.00** | 1.00 | 1.00 | **1.00** | 1.00 | 1.00 |
|  |  |  |  |  |  |  |
| **d) additional confounder: Proportion of LSOA occupied by gardens (replacing average garden size variable)** | | | | | | |
| Average garden size | **Model One:** | |  | **Model Two:** | |  |
|  | adjusted prevalence ratio | CI - upper | CI - lower | adjusted prevalence ratio | CI - upper | CI - lower |
| 1 (smallest) | **1.11** | 1.10 | 1.12 | **1.12** | 1.11 | 1.13 |
| 2 | **1.07** | 1.06 | 1.08 | **1.08** | 1.07 | 1.09 |
| 3 | **1.05** | 1.04 | 1.05 | **1.04** | 1.03 | 1.05 |
| 4 | **1.03** | 1.02 | 1.03 | **1.02** | 1.01 | 1.03 |
| 5 (largest) | **1.00** | 1.00 | 1.00 | **1.00** | 1.00 | 1.00 |
